# Validation of biomarkers for neonicotinoid exposure in *Folsomia candida* under mutual exposure to diethyl maleate

**DOI:** 10.1007/s11356-023-28940-9

**Published:** 2023-08-05

**Authors:** Ruben Bakker, Liyan Xie, Riet Vooijs, Dick Roelofs, Katja M. Hoedjes, Cornelis A. M. van Gestel

**Affiliations:** 1 Amsterdam Institute for Life and Environment (A-LIFE), Faculty of Science, Vrije Universiteit Amsterdam, De Boelelaan 1085, 1081 HV Amsterdam, The Netherlands; 2 Keygene N.V., Agro Business Park 90, Wageningen, 6708 PW The Netherlands

**Keywords:** Springtails, Neonicotinoids, Biomarkers, Glutathione-S-transferase, Diethyl maleate

## Abstract

Neonicotinoid insecticides are harmful to non-target soil invertebrates, which are crucial for sustainable agriculture. Gene expression biomarkers could provide economic and high-throughput metrics of neonicotinoid exposure and toxicity to non-target invertebrates. Thereby, biomarkers can help guide remediation efforts or policy enforcement. Gene expression of *Glutathione S-Transferase 3* (*GST3*) has previously been proposed as a biomarker for the neonicotinoid imidacloprid in the soil ecotoxicological model species *Folsomia candida* (Collembola). However, it remains unclear how reliably gene expression of neonicotinoid biomarkers, such as *GST3*, can indicate the exposure to the broader neonicotinoid family under putative GST enzymatic inhibition. In this work, we exposed springtails to two neonicotinoids, thiacloprid and imidacloprid, alongside diethyl maleate (DEM), a known GST metabolic inhibitor that imposes oxidative stress. First, we determined the influence of DEM on neonicotinoid toxicity to springtail fecundity. Second, we surveyed the gene expression of four biomarkers, including *GST3*, under mutual exposure to neonicotinoids and DEM. We observed no effect of DEM on springtail fecundity. Moreover, the expression of *GST3* was only influenced by DEM under mutual exposure with thiacloprid but not with imidacloprid. The results indicate that *GST3* is not a robust indicator of neonicotinoid exposure and that probable GST enzymatic inhibition mediates the toxicity of imidacloprid and thiacloprid differentially. Future research should investigate biomarker reliability under shifting metabolic conditions such as provided by DEM exposure.

**Supplementary Information:**

The online version contains supplementary material available at 10.1007/s11356-023-28940-9.

## Introduction

Currently, remediation efforts and policy enforcement for soil pollution rely on the chemical screening of soil samples, which is a laborious and expensive process. However, chemical analysis can only provide evidence for the presence of contaminants, not their toxicity to non-target invertebrates. In contrast, gene expression biomarkers can provide metrics of exposure to or the toxicity of soil pollutants, such as pesticides, to soil invertebrates, even under synergistic interactions in mixtures with other pollutants (Fontanetti et al. 2011; Shi et al. 2017). Additionally, biomarkers can serve as an inexpensive and high-throughput tool to screen soil samples in environmental biomonitoring (Fontanetti et al. 2011).

Neonicotinoids are harmful to non-target invertebrates, which play a crucial role in sustainable agriculture, including pollinators (Pisa et al. 2014) and soil invertebrates (de Lima e Silva et al. 2017, 2020). As such, it is imperative to develop biomarkers that indicate the exposure to these insecticides. One potential source of candidate biomarkers for neonicotinoid exposure are the genes involved in the biotransformation and detoxification of xenobiotic substances. The biotransformation pathway comprises three phases: I oxidation, II conjugation, and III excretion. Glutathione S-transferases (GSTs) are among the major enzymes involved in phase II biotransformation, which conjugate phase I metabolites to enable further excretion but also contribute to negating oxidative stress by reducing free radicals produced in phase I (Salinas and Wong 1999). Previous research has identified *Glutathione-S-Transferase 3* (*GST3)* as a potential gene expression biomarker for the neonicotinoid imidacloprid in the ecotoxicological model species *Folsomia candida* (Collembola), exposed on a plaster of Paris substrate moistened with an imidacloprid solution (Sillapawattana and Schäffer 2017). However, marked differences exist between the toxicity of individual neonicotinoids to non-target invertebrates as distinct molecular mechanisms mediate their toxicity (Buszewski et al. 2019). For example, two neonicotinoids with large differential toxicity to the fecundity and survival of springtails are imidacloprid and thiacloprid (de Lima e Silva et al. 2017, 2020; 2021). In various bee species, the differential toxicity of these insecticides has been attributed to a more readily biotransformation of thiacloprid compared to imidacloprid (Beadle et al. 2019; Manjon et al. 2018). Therefore, in order to apply *GST3* as a biomarker for neonicotinoid exposure, its gene expression should be a reliable indicator for multiple neonicotinoids and, in particular, for imidacloprid and thiacloprid.

Neonicotinoid toxicity is partially mediated by GST enzyme activity, although the exact mechanism remains unclear (Sillapawattana and Schäffer 2017). Upon exposure to increasing concentrations of the neonicotinoid imidacloprid in plaster of Paris, a depletion of the GSH pool in *F. candida* was observed by Sillapawattana and Schäffer (2017). Three possible mechanisms of GST involvement in neonicotinoid detoxification are (1) GST enzymes conjugate GSH with imidacloprid, i.e., sequestrate imidacloprid directly; (2) GST enzymes conjugate phase I metabolites of imidacloprid; and (3) GST enzymes negate the oxidative stress caused by imidacloprid toxicity or metabolism. We wish to add a fourth possibility of (4) a combination of these mechanisms. The involvement of GST in neonicotinoid metabolism differs between species. In rats, GST performs the conjugation of phase I metabolites of neonicotinoids with GSH (2) (Tomizawa and Casida 2003). However, in the honey bee, only negation by GSTs of oxidative stress caused by neonicotinoid biotransformation was observed (3) (Iwasa et al. 2004; Li et al. 2017). A reliable indicator of neonicotinoid exposure indicates the level of neonicotinoid toxicity. In order to validate *GST3* as a reliable biomarker, we therefore tested its responsiveness to neonicotinoid exposure in comparison to other biomarkers.

For this work, we selected four biomarkers in three categories: (1) GST enzymes; (2) direct targets of neonicotinoids; and (3) general oxidative stress response genes. Representing the first category, *GST3* was chosen as it was previously proposed in *F. candida* biomarker research for neonicotinoid and cadmium exposure (Nakamori et al. 2010; Sillapawattana and Schäffer 2017). The second category was represented by *nicotinic Acetylcholine receptor subunit alpha 1* (*nAchR*), which is part of the neonicotinoid target receptor and previously described as a biomarker for neonicotinoid exposure (Bakker et al. 2022). The third category was represented by *Heat Shock Protein 70 (HSP70)*, responsible for the refolding of proteins, and *Vitellogenin Receptor (VgR)*, involved in egg yolk protein production (King and Macrae 2015; Perez and Lehner 2019; Seehuus et al. 2006). Both genes have been proposed as biomarkers for pesticide exposure in the honey bee and *F. candida* (Bakker et al. 2022; Christen et al. 2018; Christen and Fent 2017). Heat Shock Proteins and Vitellogenin have been associated with the general oxidative stress response in the honey bee caused by the metabolism of nicotinoid-like substances (Rand et al. 2015). Heat shock proteins refold proteins and vitellogenin has antioxidant properties, both mitigating the damage caused by the action of free oxygen radicals or reactive oxygen species (ROS) (King and Macrae 2015; Perez and Lehner 2019; Seehuus et al. 2006). To be a reliable indicator of neonicotinoid exposure, *GST3* (category 1) should closely follow the expression of *nAchR* (category 2) and less closely that of the general stress response genes *HSP70* and *VgR* (category 3).

In order to validate *GST3* as a biomarker for neonicotinoid exposure, we investigated the expression of *HSP70*, *GST3*, and the *VgR*, as biomarkers under the mutual exposure of imidacloprid or thiacloprid with diethyl maleate (DEM), which depletes cellular GSH levels, thereby limiting GST-mediated negation of oxidative stress (Bernard and Philogène 1993; Costa and Murphy 1986; Plummer et al. 1981). The metabolic inhibitor DEM is routinely used to study the effects of GST enzymes on pesticide toxicity (see, e.g., Wu et al. 2007). By choosing DEM over pollutants found in the soil, we ensure the observed effects on gene expression are the result of probable GST inhibition and not of additional toxic effects with unknown molecular mechanisms. In this way, DEM serves as a “stress-test” that can provide evidence for the role of GST-mediated detoxification in neonicotinoid toxicity and its reliability as a biomarker for indicating neonicotinoid exposure. By exposing the springtails in soil, their natural habitat, we tried to avoid the possible influence of unfavorable environmental conditions they may encounter on a plaster of Paris substrate. Additionally, the target receptor of neonicotinoids, *nicotinic Acetylcholine Receptor (nAchR),* was also tested as it was proven to be a prominent neonicotinoid biomarker in previous studies on the honey bee (Christen et al. 2016) and *F. candida* (Bakker et al. 2022). First, we determined the influence of DEM on neonicotinoid toxicity to springtail fecundity. Second, we surveyed the gene expression of four biomarkers under mutual exposure of the two neonicotinoids with DEM.

## Methods

### Animals, chemicals, and test soil

*Folsomia candida* were obtained from inhouse cultures at the Vrije Universiteit Amsterdam, Amsterdam Institute for Life and Environment (A-LIFE) (Berlin strain). Rearing and age synchronization of the individuals have been described by de Lima e Silva et al. (2017; 2020).

Imidacloprid and thiacloprid, both > 98% purity, were provided by Bayer CropSciences, Monheim, Germany. Diethyl maleate (DEM; > 98% purity) was obtained from Sigma-Aldrich, the Netherlands.

The LUFA 2.2 test soil originated from Lufa, Speyer, Germany. The soil attributes, reported by the supplier, were total organic carbon content 2.1%, water holding capacity (WHC) 46.5% (w/w), and soil pH 5.5 (0.01 M CaCl_2_). DEM was directly dissolved in acetone, imidacloprid in ultra-pure water. Thiacloprid was first dissolved in acetone amounting to 3% of the total stock solution volume consisting of ultra-pure water. Both stock solutions were stirred overnight at 300 rounds per minute, in the dark and at room temperature.

For all treatments, 10% of the dry soil per treatment was completely inundated by acetone, with the desired concentration of DEM, in a glass jar and stirred every half hour for 2 h, in the dark, covered with aluminum foil. Then, the soil was left overnight in a fume hood to allow complete evaporation of the acetone. The remaining soil was added, mixed, and moistened to 50% of the WHC, either by adding water (for the DEM only exposures) or the neonicotinoid solutions (for the mixtures), and mixed again. For the single neonicotinoid exposures, soils were directly spiked with stock solutions and moistened to 50% of the WHC. All test soils were prepared the day before starting springtail exposures. The concentrations for the DEM single exposure were 0, 1.1, 3.3, 10, 30, and 90 mg kg^−1^ dry soil. The concentrations for mutual exposure with neonicotinoids were 0, 1, and 6 mg DEM kg^−1^ dry soil; 0, 0.25, 0.5, 1, 2, 4, 8, and 16 mg thiacloprid kg^−1^ dry soil; and 0, 0.05, 0.1, 0.2, 0.4, 0.8, and 1.6 mg imidacloprid kg^−1^ dry soil. For the mutual exposure tests, DEM concentrations were roughly equal to the effect concentrations (EC_x_) reducing the number of juveniles by 0, 1, and 25%. Water controls were included in all tests by moistening LUFA 2.2 soil with demineralized water to 50% of its WHC and mixed thoroughly.

To determine the accuracy of pesticide application, 3–5 g portions of test soil were stored at − 20 °C and sent to Groen Agro Control, Delfgauw, the Netherlands. Here, the pesticide soil concentrations were measured following a certified protocol and with a detection limit of 0.01 mg kg^−1^.

For the gene expression assay, concentrations of 0, 10, and 20 mg DEM kg^−1^ dry soil were combined with 0 (control), 0.1, 0.2, and 0.4 mg imidacloprid kg^−1^ dry soil or 0 (control), 0.5, 1, and 2 mg thiacloprid kg^−1^ dry soil. The neonicotinoid concentrations represented roughly the neonicotinoid EC_10_, EC_25_, and EC_50_ for effects on springtail fecundity from earlier studies in our laboratory (de Lima e Silva et al. 2021; 2020).

### Toxicity tests

The toxicity tests followed OECD guideline 232 for Collembolan reproduction toxicity testing in soil (OECD 2016). Briefly, 10 adult *F. candida* were exposed in 30 g of test soil at a room temperature of 20 ± 1 ℃, relative air humidity of 75%, and a 16:8 light–dark regime. The springtails were fed ad libitum baker’s yeast (Algist Bruggeman N.V., Ghent, Belgium), and the soil water content was maintained throughout the experiment. We made two deviations from the OECD 232 guideline: the duration of the test was shortened from 28 to 21 days, and the initial age of the animals was 21–23 days (instead of 10–12 days). These changes were made to facilitate comparison between the reproduction tests and gene expression assays. Adults of 21–23 days old were also used in the gene expression assay (next paragraph). The exposure duration was shortened because *F. candida* reaches sexual maturity after about 18 days, so after about 7 days in the standard 28-day test (OECD 2016). By shortening the exposure duration, the *F. candida* reproduction toxicity tests are better comparable to other experiments using 10–12-day-old juveniles (OECD 2016) as the duration of exposure of the adult phase is the same, i.e., 21 days. At the end of the toxicity tests, the samples were emptied into plastic beakers and their contents waterlogged using tap water, stirred gently, and left to rest for at least 5 min to allow all animals to come floating to the surface. The surface was photographed by a Nikon Coolpix P510, and the *F. candida* adults and juveniles were counted using Image-J based software Fiji (v. 1.52p) with the Cell Counter plugin (Kurt de Vos, version from 2010).

### Gene expression assay

Roughly 30 age-synchronized, 21–23-day-old, *F. candida*, were exposed for 48 h in 30 g of test soil; see paragraph on test soil. No food was added to the test soil. Then, the samples were water logged, left to rest for 5 min, and the springtails were scooped into containers using a fine mesh. The springtails were transferred to 1.5-ml reaction tubes with an aspirator and snap frozen in liquid nitrogen. Springtail RNA was extracted using the SV Total RNA extraction kit (Promega, USA), according to the manufacturer’s guidelines. Total RNA purity and quantity were measured spectrophotometrically using a Nanodrop (Thermo-Fisher, the Netherlands). A 1% agarose gel containing 0.5% ethidium bromide was used to check the quality of the RNA. Following the manufacturer’s instructions, approximately 500 ng of RNA was reverse-transcribed into cDNA using the Promega MML-V reverse transcriptase kit. For one out of seven samples, a no cDNA sample was created by excluding reverse transcriptase from the reactions in order to verify the absence of DNA contamination. Using BIO-RAD 96-well plates and Cyber Green mix, quantitative PCR (qPCR) analysis was carried out on a CFX Connect Real-Time PCR Detection System (BIO-RAD, USA). Each sample was run in duplicate including no sample controls.

The selected target genes were *nicotinic Acetylcholine Receptor-subunit alpha1* (nAchR), the binding site of neonicotinoids; *Heat Shock Protein 70* (*HSP70*), involved in protein refolding after endured stressors, such as oxidative stress (King and Macrae 2015); *Vitellogenin Receptor (VgR*), which activation leads to egg yolk production and transport, but has been linked to oxidative stress response as well (Perez and Lehner 2019; Seehuus et al. 2006); *Glutathione-S-Transferase 3* (*GST3*), which negates oxidative stress by reducing reactive compounds; and the reference genes *Tyrosine 3-Monooxygenase* (YWHAZ) and *Eukaryotic Transcription Initiation Factor 1A* (ETIF). The primer sets for nAchR (Bakker et al. 2022), *YWHAZ*, *ETIF*, *HSP70*, and *VgR* were taken from earlier work (de Boer et al. 2011; Roelofs et al. 2012). The *GST3* primer set was custom made for this work with Primer BLAST (Ye et al. 2012), based on the *GTS3* gene described by Nakamori et al. (2010) and Sillapawattana and Schäffer (2017). For the primer sequences and efficiencies, see Table S-1 in the “Supplementary information.” All measurements were performed in duplicate, and if results differed by half a threshold cycle (Cq), they were discarded and the measurements repeated. If results for any reference gene differed by half a threshold cycle (Cq), the measurements were repeated for all primer sets for that sample.

### Data analysis

All statistical analyses were used the *R* programming language v4.0.0 (R Core Team 2019). Three parameter logistic concentration–response curves were fitted over the number of adult or juvenile *F. candida* to calculate the effect concentrations for survival and reproduction, respectively, using the R-package *drc* v3.0–1 (Ritz et al. 2015). Models constrained and unconstrained in their EC_50_-estimate, i.e., concentration reducing juvenile counts by 50%, were compared using the loglikelihood ratio test. Graphics were generated using *ggplot2* v3.3.5 throughout this work (Wickham 2016).

Normalized gene expression values were obtained using the qPCR accompanying software CFX manager (BIO-RAD, USA) by creating a gene study and exporting the untransformed values. Briefly, this software output presents the gene expression values as follows. Gene expression in qPCR is defined as the number of cycles necessary to reach the threshold (Cq), in our case 1000 arbitrary light intensity units. All expression values are adjusted by the primer-set efficiencies (E), see Table S-1. Per sample, all expression values were subtracted by the lowest expression value. Target gene expression per sample was then divided by the average expression of the two reference genes. The resulting gene expression values are a ratio and do not adhere to homogeneity and independence of variance. Therefore, we applied log2-transformation to the gene expression values.

For each primer set, a generalized additive model (GAM) was fitted over the log2-transformed normalized gene expression values using the R-package *mgcv* v1.8–37 (Wood 2011). The null model considered only neonicotinoid influence on gene expression, Eq. 1, and the alternative model also included the influence of DEM, Eq. 2.

1 $$E={{g}^{-1}(\beta }_{0}+\sum\nolimits_{j=1}^{{k}_{1}}{\beta }_{1}{s}_{j}{(X}_{j}))$$

2 $$E={{g}^{-1}(\beta }_{0}+\sum\nolimits_{j=1}^{{k}_{1}}{\beta }_{j}{s}_{j}{(x}_{j})+ \sum\nolimits_{p=1}^{{k}_{2}}{\beta }_{p}{s}_{p}{(X}_{p}))$$

in which *E* is the expected value of the log2-normalized expression values, g^−1^ the inverse linkage function, *β*_0_ the intercept, *β*_j_ and *β*_p_ the coefficients for neonicotinoid (j) and DEM exposure (p), and *s*_j_ and *s*_p_ smooth terms for neonicotinoid (j) and DEM exposure (p) with *k* the basis size, respectively.

Basis size (*k*_x_) for the neonicotinoid smooth term (*k*_1_) was set to four, and for the DEM smooth term (*k*_2_), it was set to three, i.e., the maximum size for this experimental design. Gaussian error distribution of the residuals was assumed; thin-plate regression splines and restricted maximum likelihood (REML) were used to fit the models. Models were compared using an *F*-test of their fits, and the alternative model was accepted when *p* ≤ 0.1. Adherence of homogeneity of residuals was visually checked by histogram frequency plot and quantile–quantile plots.

## Results and discussion

### Soil concentrations

Neonicotinoid concentrations were measured in test soil spiked with concentrations around the EC_50_ for effects on springtail fecundity for imidacloprid (0.2 and 0.4 mg kg^−1^ dry soil) and thiacloprid (1 mg kg^−1^ dry soil). Measured imidacloprid concentrations were on average 87% (SD 6.7%, *n* = 6) of the nominal ones (Table S-2). The measured and nominal concentrations of thiacloprid were highly similar, with an average recovery of 98% (SD 12.5%, *n* = 3). Imidacloprid degraded to 85% of its measured concentration between the onset and the end (day 21) of the toxicity test. Thiacloprid degraded almost completely to only 2.5% of its initial measured concentration within the 21-day test period. Because of the high recovery of both test compounds at the start of the exposures, all data are based on the nominal concentrations.

### Test validity

With the exception of two groups, all control groups that did not receive neonicotinoid treatments, so including those treated with 1 or 6 mg DEM kg^−1^ dry soil, met the validity criteria of OECD guideline 232 (OECD 2016), namely, mean juvenile count > 100, variation in control juvenile counts < 30%, adult survival > 80% (see Table S-3). For the single diethyl maleate (DEM) exposures, the variation in the number of juveniles was 36% in the control, and for the thiacloprid toxicity test, it was 32% in the 6 mg DEM kg^−1^ dry soil control group (i.e., without thiacloprid). A high variation in the number of juveniles is common in *F. candida* reproduction tests (Crouau and Cazes 2003), and the higher variation was not associated with any particular treatment across the toxicity tests with THI, IMI and DEM. We therefore conclude that the springtail health was good at the start of the toxicity tests and did not bias the results.

### Effects of DEM on springtail fecundity and mortality

**Fig. 1:**
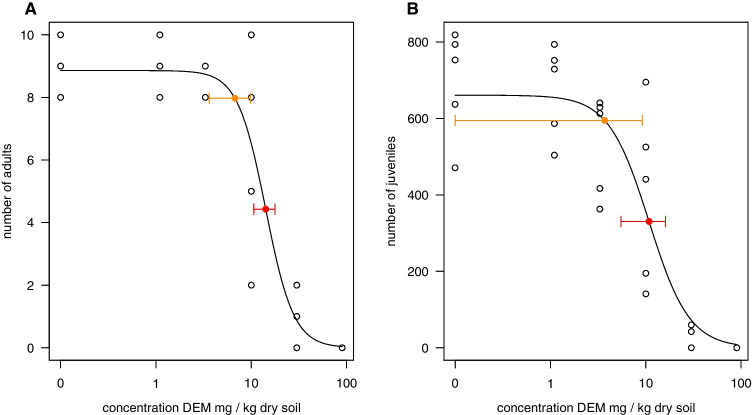
The effects of diethyl maleate (DEM) on the survival (**A**) and reproduction (**B**) of *Folsomia candida* after 21 days exposure in LUFA 2.2 soil. The juvenile and adult counts are shown as circles on the panels; the solid line shows the fit of a three-parameter logistic model. Concentrations affecting survival and reproduction by 10% and 50%, LC_10_ and LC_50_ (**A**) or EC_10_ and EC_50_ (**B**) respectively, are shown as orange and red. Whiskers show the 95% confidence interval estimators as obtained using the delta method

DEM reduced the adult springtail survival by 1, 10, and 50% at 2.99, 6.73, and 14.2 mg DEM kg^−1^ dry soil, i.e., the LC_1_, LC_10_, and LC_50_, respectively (see Fig. 1 and Table 1). DEM, as a single compound, reduced the number of juveniles by 1% (EC_1_) at 1.15 mg kg^−1^ dry soil and had an estimated EC_10_ of 3.7 mg kg^−1^ dry soil and EC_50_ of 10.9 mg kg^−1^ dry soil (Table 1). The DEM concentrations affecting survival (LC_x_) and fecundity (EC_x_) did not differ much, as also shown by the similar concentration–response curves (Fig. 1) and overlapping 95% confidence intervals (Table 1). The close effect concentrations for survival and fecundity suggest that DEM reduces fecundity as a direct consequence of reduced survival and elicits little sublethal toxic effect.

**Table 1:** Toxicity of diethyl maleate (DEM) on reproduction (EC_x_) (A) and survival (LC_x_) (B) and its effect on the toxicity of imidacloprid and thiacloprid to the reproduction of *Folsomia candida* after 21 days of exposure in LUFA 2.2 soil

(A)	Exposure	DEM (mg kg^−1^ dry soil)	EC_1_ (mg kg^−1^ dry soil)	EC_10_ (mg kg^−1^ dry soil)	EC_50_ (mg kg^−1^ dry soil)
	Imidacloprid	0	0.02 (0:0.05)	0.08 (0.03:0.12)	0.24 (0.19:0.29)
		1	0.06 (0.01:0.12)	0.15 (0.08:0.22)	0.32 (0.26:0.37)
		6	0.02 (0:0.04)	0.07 (0.03:0.11)	0.24 (0.17:0.30)
	Thiacloprid	0	0.09 (− 0.02:0.19)	0.44 (0.14:0.74)	1.94 (1.35:2.53)
		1	0.12 (− 0.02–0.19)	0.52 (0.18:0.85)	1.96 (1.41:2.50)
		6	0.20 (− 0.02:0.43)	0.72 (0.28:1.16)	2.28 (1.71:2.86)
	DEM	NA	1.15 (− 1.90:4.21)	3.70 (− 1.75:9.16)	10.8 (5.49:16.1)
(B)			LC_1_ (mg kg^−1^ dry soil)	LC_10_ (mg kg^−1^ dry soil)	LC_50_ (mg kg^−1^ dry soil)
	DEM	NA	2.99 (0.31:5.66)	6.73 (3.63:9.83)	14.18 (10.61:17.75)

EC_1_, EC_10_, and EC_50_ are effective concentrations reducing juvenile numbers by 1, 10, and 50% compared to the control, respectively (see part A). LC_1_, LC_10_, and LC_50_ are lethal concentrations reducing adult survival by 1, 10, and 50%, respectively (see part B). Values in parenthesis are 95% confidence intervals calculated using the delta method

### Effects of DEM on neonicotinoid toxicity to springtail fecundity

**Fig. 2:**
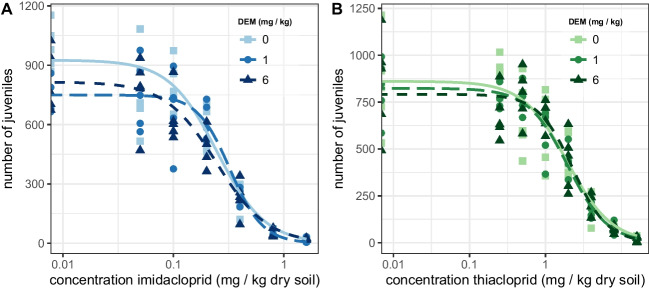
The effect of diethyl maleate (DEM) on the toxicity of the neonicotinoids imidacloprid (**A**) and thiacloprid (**B**) to the fecundity of the springtail *Folsomia candida* after 21 days exposure in LUFA 2.2 soil. Juvenile counts per sample are shown as markers. Lines show the fit to the data of the three-parameter logistic model. Line and marker type vary per level of DEM: solid lines and squares for 0 mg kg^−1^ dry soil, long-dashed lines and circles for 1 mg DEM kg^−1^ dry soil, and short-dashed lines and triangles for 6 mg DEM kg^−1^ dry soil

The neonicotinoids thiacloprid and imidacloprid did not cause sufficiently high adult springtail mortality at their highest test concentrations to allow fitting concentration–response curves; therefore, no LC_50_ values could be calculated. Thiacloprid reduced juvenile counts with EC_1_, EC_10_, and EC_50_ values of 0.09, 0.44, and 1.94 mg kg^−1^ dry soil, respectively (Table 1). The EC_x_ values were not affected by DEM exposure as their 95% confidence intervals were overlapping. The concentration–response curves for each level of DEM were overlapping or at least adjacent (see Fig. 2). Also, the EC_50_ estimates did not differ between the levels of DEM exposure (*p* = 0.66, loglikelihood ratio test). Combined, the results indicate no influence of DEM exposure on the toxicity of thiacloprid to springtail fecundity.

In the absence of DEM, the estimated EC_1_, EC_10_, and EC_50_ values for the effects of imidacloprid on springtail fecundity were 0.02, 0.08, and 0.24 mg kg^−1^ dry soil, respectively (Table 1). The EC_x_ estimates for the effects of imidacloprid on springtail fecundity showed overlapping 95% confidence intervals between the different levels of DEM exposure (see Table 1). The concentration–response curves (Fig. 2) largely overlapped for intermediate until high concentrations of imidacloprid, i.e., 0.1 mg kg^−1^ dry soil and above, indicating similar effects of imidacloprid on springtail fecundity independent of DEM exposure. The comparison of the EC_50_ values between the levels of DEM showed moderate effects of DEM on imidacloprid toxicity (*p* = 0.07, loglikelihood ratio test). However, as the 95% confidence intervals of the EC_x_ values largely overlapped, we conclude that DEM also did not alter the toxicity of imidacloprid to springtail fecundity.

Our data indicate that when applied in mixtures, DEM did not alter the toxicity of either thiacloprid or imidacloprid. Most research investigating the influence of DEM exposure on neonicotinoid toxicity has been performed on neonicotinoid-resistant insect pests with the aim to provide evidence that increased GST enzymatic activity contributes to neonicotinoid resistance. Imidacloprid is the most well-studied neonicotinoid in this body of research. No influence of DEM on the survival of the neonicotinoid-susceptible strains of the brown planthopper (*Nilaparvata lugens*), the melon/cotton aphid (*Aphis gossypii*), the sweet potato whitefly (*Bemisia tabaci*), or the English grain aphid (*Sitobion avenae*) under imidacloprid exposure was found (Bao et al. 2016; Salehi-Sedeh et al. 2020; Seyedebrahimi et al. 2016; Zhang et al. 2020). DEM also did not influence the toxicity of the neonicotinoid dinotefuran to the survival of the melon/cotton aphid *A. gossypii* (Chen et al. 2020). Lastly, no influence was found of DEM on the toxicity of the neonicotinoid acetamiprid to the honey bee (*Apis mellifera)* and the sweet potato whitefly (*B. tabaci*) (Feng et al. 2010; Iwasa et al. 2004). Therefore, our results are in line with previous findings that GST inhibition by DEM does not increase the toxicity of neonicotinoids to insects and related organisms like springtails.

### Gene expression responses to DEM and neonicotinoids

For gene expression responses, the adherence of the generalized additive model fit to homogeneity was confirmed by inspecting frequency and quantile–quantile plots (see Figure S-1 and Figure S-2). From the residuals, no noteworthy deviation from homogeneity was found.

**Fig. 3:**
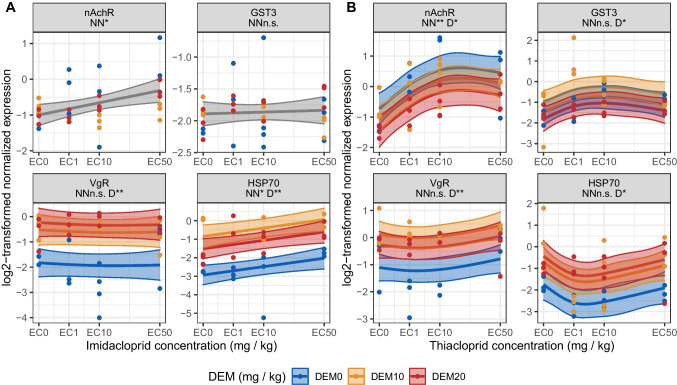
The influence of diethyl maleate (DEM) on the gene expression of the springtail *Folsomia candida* induced by 48-h exposure to the neonicotinoids imidacloprid (**A**) or thiacloprid (**B**) in LUFA 2.2 soil. Each panel represents the results of one gene, the abbreviations are listed in the portrait headers: *nicotinic Acetylcholine Receptor subunit alpha1* (*nAchR*), *Glutathione-s-Transferase 3* (*GST3*), *Vitellogenin Receptor* (*VgR*), and *Heat Shock Protein 70* (*HSP70*). Below the names are the significance levels of the generalized additive models (GAMs) smooth terms of neonicotinoid (NN) and DEM (D). Significance levels of the smooth terms are depicted by the following symbols: *p* > 0.1 “N.S”; *p* ≤ 0.1 “.”; *p* ≤ 0.05 “*”; and *p* ≤ 0.01 “**”. GAM mean functions are shown in solid lines, the 95% confidence intervals as outlined transparent bands, and dots depict the log2-transformed normalized expression values. Each level of DEM exposure is shown as a separate color, i.e., blue, orange, and red for 0, 10, and 20 mg DEM kg^−1^ dry soil, respectively. Mean function and confidence interval outlined bands are shown in gray when the influence of DEM was not included in the GAM model fit

Neonicotinoid exposure up to concentrations equal to the EC_50_, so high enough to evoke phenotypic effects, did not influence the gene expression of *VgR* and *GST3* (see Fig. 3). Both neonicotinoids enhanced the expression of *nAchR*, and imidacloprid also enhanced the expression of *HSP70*. The gene expression patterns upon thiacloprid exposure are different from those for imidacloprid exposure by one key aspect: imidacloprid induced gene expression in a concentration-dependent manner, while gene expression upon thiacloprid exposures was at maximum or minimum at intermediate exposure levels and returned to control levels at high exposure concentrations (see Fig. 3). This agrees with our previous findings on *F. candida* in LUFA 2.2 soil*,* also showing enhanced gene expression of *nAchR* following exposure to both neonicotinoids and non-linear gene expression patterns of various biomarkers upon thiacloprid exposure (Bakker et al. 2022). Our previous findings and the results of this work therefore indicate that different molecular mechanisms mediate the toxicity of the two neonicotinoids at higher exposure levels.

At the EC_0_ exposure of both neonicotinoids, DEM enhanced *HSP70* and *VgR* gene expression above control conditions as indicated by non-overlapping confidence intervals of the GAM fits and the measurements indicated by the dots on the panels in Fig. 3. Both biomarkers are associated with the oxidative stress response (Perez and Lehner 2019; Wu et al. 2021; King and Macrae 2015; Seehuus et al. 2006). This finding supports the notion that DEM exposure alone increases oxidative stress in *F. candida* and provides evidence for the successful absorption by *F. candida* of DEM and subsequent GST inhibition as has been established in the scientific literature for other organisms across the animal kingdom, from fruit flies to humans (Bernard and Philogène 1993; Costa and Murphy 1986; Plummer et al. 1981).

DEM increased the expression of *HSP70* and *VgR* under the mutual exposure with both neonicotinoids (see Fig. 3). For both genes, expression was increased by DEM exposure compared to neonicotinoid exposure in the absence of DEM. The extent by which DEM induced the expression of *VgR* and *HSP70* was similar between the two levels of DEM as indicated by overlapping confidence intervals (see Fig. 3). The influence of DEM on *nAchR* and *GST3* was different in the mutual exposures with the two neonicotinoids. Under mutual exposure with thiacloprid, DEM decreased the expression of *nAchR* and altered the expression of *GST3*. *GST3* expression was increased at 10 mg DEM kg^−1^ dry soil and decreased by 20 mg DEM kg^−1^ dry soil under co-exposure with thiacloprid, showing a non-linear response of *GST3* to DEM exposure. Hence, the results indicate that thiacloprid exerts a stronger oxidative stress response compared to imidacloprid because the gene expression of all four biomarkers was altered only following mutual exposure to DEM with thiacloprid.

As indicated in the “Introduction,” there are four possible mechanisms of GST enzyme involvement in neonicotinoid detoxification: (1) conjugating GSH with imidacloprid, (2) conjugating phase I metabolites of imidacloprid, (3) negating oxidative stress caused by imidacloprid toxicity or metabolism, and (4) a combination of these mechanisms. Sillapawattana and Schäffer (2017) observed *GST3* upregulation and increased GST enzymatic activity in *F. candida* exposed to imidacloprid on a plaster of Paris substrate, which confirms the first three mechanisms in this work. We observed no upregulation of the *GST3* by the two neonicotinoids; therefore, our findings do not support these mechanisms. Our findings suggest that oxidative stress was increased by DEM exposure. Support for this comes from the expression of *VgR* and *HSP70*. Both genes perform a diverse set of functions (Perez and Lehner 2019; Wu et al. 2021) and have both been linked to the general oxidative stress response (King and Macrae 2015; Seehuus et al. 2006). Various GSTs are encoded by *F. candida*, and it is possible that other GSTs are involved in the biotransformation of neonicotinoids or its metabolites and respond to neonicotinoid exposure. It may also be possible that the exposure conditions on the plaster of Paris substrate used by Sillapawattana and Schäffer (2017) were different from those in the LUFA 2.2 soil, further adding to the differences in the response of the springtails to imidacloprid. Future research into the expression of these GSTs under neonicotinoid exposure of springtails is needed to refute or support the different mechanisms. For the biomarker *GST3*, we found that its gene expression was no reliable indicator of neonicotinoid exposure in *F. candida*, neither for imidacloprid nor for thiacloprid.

The gene-expression results suggest that DEM exposure increases oxidative stress and altered the gene-expression patterns of all candidate biomarkers under mutual exposure with at least one neonicotinoid. However, for both neonicotinoids, no effects of DEM exposure were found on neonicotinoid toxicity to *F. candida* fecundity. Toxicity is multifaceted and can relate to, among others, behavior, reproduction, or survival. A possible explanation for the observed effect of DEM on gene expressions and not fecundity could be that DEM has little sublethal effects and, hence, has fewer interaction effects with neonicotinoids affecting reproduction. Secondly, gene expression is a more specific and sensitive metric of pesticide exposure compared to fecundity and precedes effects observed on the phenotype. Therefore, gene expression effects can be observed not (yet) affecting downstream phenotypic measures of toxicity.

The effects of toxicants on gene expression is diverse, and, hence, multiple biomarkers have to be combined in order to provide a reliable read-out of pesticide soil pollution (Lionetto et al. 2019). No suitable selection of candidate biomarkers has been identified in this study to indicate neonicotinoid exposure. However, the aim of this study was to investigate the influence of oxidative stress on biomarker reliability, not to provide a comprehensive panel of biomarkers. In our previous study, we found that the expressions of *nAchR* and *Sodium-Coupled Monocarboxylate Transporter* (*SMCT*) *1* were reliable indicators of neonicotinoid exposure when used in combination (Bakker et al. 2022). Therefore, future studies should attempt to incorporate novel biomarkers into a panel that includes *nAchR* and *SMCT* for neonicotinoid exposure. The current work provides a tool, i.e., mutual exposure with DEM, for testing the reliability of the resulting biomarkers under varying oxidative stress conditions.

## Conclusion

Our goal was to investigate the reliability of *Folsomia candida* (springtail) biomarkers as indicators of neonicotinoid exposure in soil under increased oxidative stress exerted by co-exposure to DEM, a metabolic inhibitor of GST enzymes. In particular, we surveyed the previously described imidacloprid biomarker *GST3.* We found that DEM did not influence the toxicity of the neonicotinoids imidacloprid and thiacloprid to springtail fecundity. Moreover, both neonicotinoids did not affect the expression of *GST3*. However, DEM exposure influenced the gene expression of *VgR* and *HSP70* under mutual exposure with both neonicotinoids. Combined, the results indicate that GST enzyme activity does not strongly mediate neonicotinoid toxicity to springtail fecundity and that the expression of *GST3* is no reliable biomarker for neonicotinoid exposure. Additionally, we observed that the gene expression of all considered candidate biomarkers was altered by DEM co-exposure, at least for one of the two neonicotinoids. This suggests that increased oxidative stress is an important factor for the reliability of biomarkers indicating neonicotinoid exposure. Therefore, our data support the hypothesis that DEM could provide a “stress-test” to study biomarker reliability under such conditions. The results of this work give insights into the influence of GST-mediated biotransformation on neonicotinoid toxicity and indicate that different molecular mechanisms mediate the toxicity of imidacloprid and thiacloprid in an important soil ecotoxicological model species.

## Supplementary Information

Below is the link to the electronic supplementary material.

Supplementary file1 (DOCX 393 KB)

## Data Availability

An online repository is available with the R-code, reproduction count data, and gene expression data, see: https://zenodo.org/record/7551557.
