# Supplementary material for: Validation of biomarkers for neonicotinoid exposure in Folsomia candida under mutual exposure to diethyl maleate
Source: Environ Sci Pollut Res Int. 2023 Aug 5;30(42):95338–47. doi: 10.1007/s11356-023-28940-9 (PMC10482762; doi:10.1007/s11356-023-28940-9)
Supplement: Supplementary file 1 — Supplementary file1 (DOCX 393 KB) [file 11356_2023_28940_MOESM1_ESM.docx]

Supplementary Information to:

# **Validation of biomarkers for neonicotinoid exposure in *Folsomia candida* under mutual exposure to diethyl maleate**

Ruben Bakker^1^, Liyan Xie^1^, Riet Vooijs^1^, Dick Roelofs^1,2^, Katja M. Hoedjes^1^, Cornelis A.M. van Gestel^1*^

^1^ Amsterdam Institute for Life and Environment (A-LIFE), Faculty of Science, Vrije Universiteit Amsterdam, De Boelelaan 1085, 1081 HV Amsterdam, The Netherlands

^2^ Keygene N.V., Agro Business Park 90, Wageningen, 6708 PW, The Netherlands

* corresponding author

Corresponding e-mail: [kees.van.gestel@vu.nl](mailto:kees.van.gestel@vu.nl)

**Table S‑1: Summary of the primer sets used in quantitative PCR to determine the effects of neonicotinoid and/or DEM exposure on the gene expression of Folsomia candida in LUFA 2.2 soil.** The name of the primer sets used, the primer direction, oligo sequence, the description of the primer set target, the target gene id according to Ensembl Metazoa release version 50 (Cunningham et al., 2019) and the efficiency at which the sets amplify the DNA fragments at each cycle shown as percentages.

| **name** | **direction** | **oligo sequence** | **description** | **gene id** | **Efficiency (%)** |
| --- | --- | --- | --- | --- | --- |
| *YWHAZ* | Forward | CCTACAAAAACGTCGTCGGTG | *Tyrosine 3-Monooxygenase* | Fcan01_06830 | 89.3 |
|  | Reverse | TGTTGCTTTCGTTCGAACC |  |  |  |
| *ETIF* | Forward | TGATTCTGGAGATCTTCGCGAG | *Eukaryotic Transcription Initiation Factor 1A* | Fcan01_13627 | 94.9 |
|  | Reverse | ACAGTGCAAAGGATTTCCCGA |  |  |  |
| *GST3* | Forward | CAACGATCTCTTTGAGCAGTGG | *Glutathione-S-Transferase 3* | Fcan01_00498 | 93.2 |
|  | Reverse | CTTCCAAGCATTTAGGCGCA |  |  |  |
| *VgR* | Forward | TGTCCCGTAGGGATGTATCTTGA | *Vitellogenin Receptor* | Fcan01_04244, Fcan01_04245 | 85.6 |
|  | Reverse | GATTGTGTTGTTGTACCCGATGAC |  |  |  |
| *HSP70* | Forward | TTGGTCGACGTAGCTCCACTCT | *Heat Shock Protein 70* | Fcan01_10020 | 98.1 |
|  | Reverse | TGGGCTTGTTTGCATGGAAT |  |  |  |
| *nAchR* | Forward | CGTGGACCAGGACAGAGAAA | *nicotinic Acetylcholine Receptor subunit alpha 1* | Fcan01_01431 | 85.6 |
|  | Reverse | TTGCAGACCCCCATAGTCTG |  |  |  |

**Table S-2: Concentrations of imidacloprid and thiacloprid in LUFA2.2 soil measured on the day of soil spiking and at the end of the 21-day exposure of *Folsomia candida* in LUFA 2.2 soil.** Recovery was calculated by dividing the measured over the nominal concentration and expressing it as a percentage. The recovery is not calculated for the samples analyzed after 21 days exposure, or if the nominal concentration was zero (shown as NA, not applicable).

| neonicotinoid | nominal concentration  (mg kg^-1^ dry soil) | day |  | measured concentration  (mg kg^-1^ dry soil) | Recoveries  (%) |
| --- | --- | --- | --- | --- | --- |
|  |  |  |  |  |  |
| Imidacloprid | 0 | 0 |  | 0 | NA |
|  | 0.2 | 0 |  | 0.18 | 90 |
|  | 0.2 | 0 |  | 0.17 | 84 |
|  | 0.2 | 0 |  | 0.19 | 96 |
|  | 0.4 | 0 |  | 0.36 | 90 |
|  | 0.4 | 0 |  | 0.31 | 78 |
|  | 0.4 | 0 |  | 0.32 | 81 |
|  | 0.4 | 21 |  | 0.28 | NA |
|  |  |  |  |  |  |
| Thiacloprid | 0 | 0 |  | 0 | NA |
|  | 2 | 0 |  | 2.04 | 102 |
|  | 2 | 0 |  | 1.68 | 84 |
|  | 2 | 0 |  | 2.16 | 108 |
|  | 2 | 21 |  | 0.05 | NA |
|  |  |  |  |  |  |

**Table S-3: Control group performance of Folsomia candida in LUFA 2.2 soil in toxicity tests with diethyl maleate (DEM) and/or the neonicotinoids imidacloprid or thiacloprid.** Reference groups were exposed to soils only treated with demineralized water, pretreated with acetone or pretreated with acetone and either 1 or 6 mg DEM kg^-1^: abbreviated as water, acetone and DEM 1 or DEM 6, respectively. Also added are the validity criteria according to the OECD guideline 232 (OECD, 2016). The reference group of DEM 6 in the thiacloprid test and water control of the DEM test are marked in bold as they did not meet these criteria with a coefficient of variance of 32 and 36 %, respectively.

| **Compounds** | **Control type** | **Mean adult mortality (%)** | **Mean juvenile count** | **Coefficient of variance (%)** |
| --- | --- | --- | --- | --- |
|  |  |  |  |  |
| Imidacloprid and DEM | water | 20 | 964 | 11 |
|  | acetone | 8 | 991 | 11 |
|  | DEM 1 | 18 | 771 | 12 |
|  | DEM 6 | 10 | 820 | 19 |
|  |  |  |  |  |
| Thiacloprid and DEM | water | 8 | 743 | 18 |
|  | acetone | 16 | 843 | 30 |
|  | DEM 1 | 6 | 822 | 19 |
|  | DEM 6 | 6 | 852 | **32** |
|  |  |  |  |  |
| DEM | water | 10 | 670 | **36** |
|  | acetone | 8 | 695 | 21 |
| OECD validity criteria |  | < 20 % | >100 | <30 |


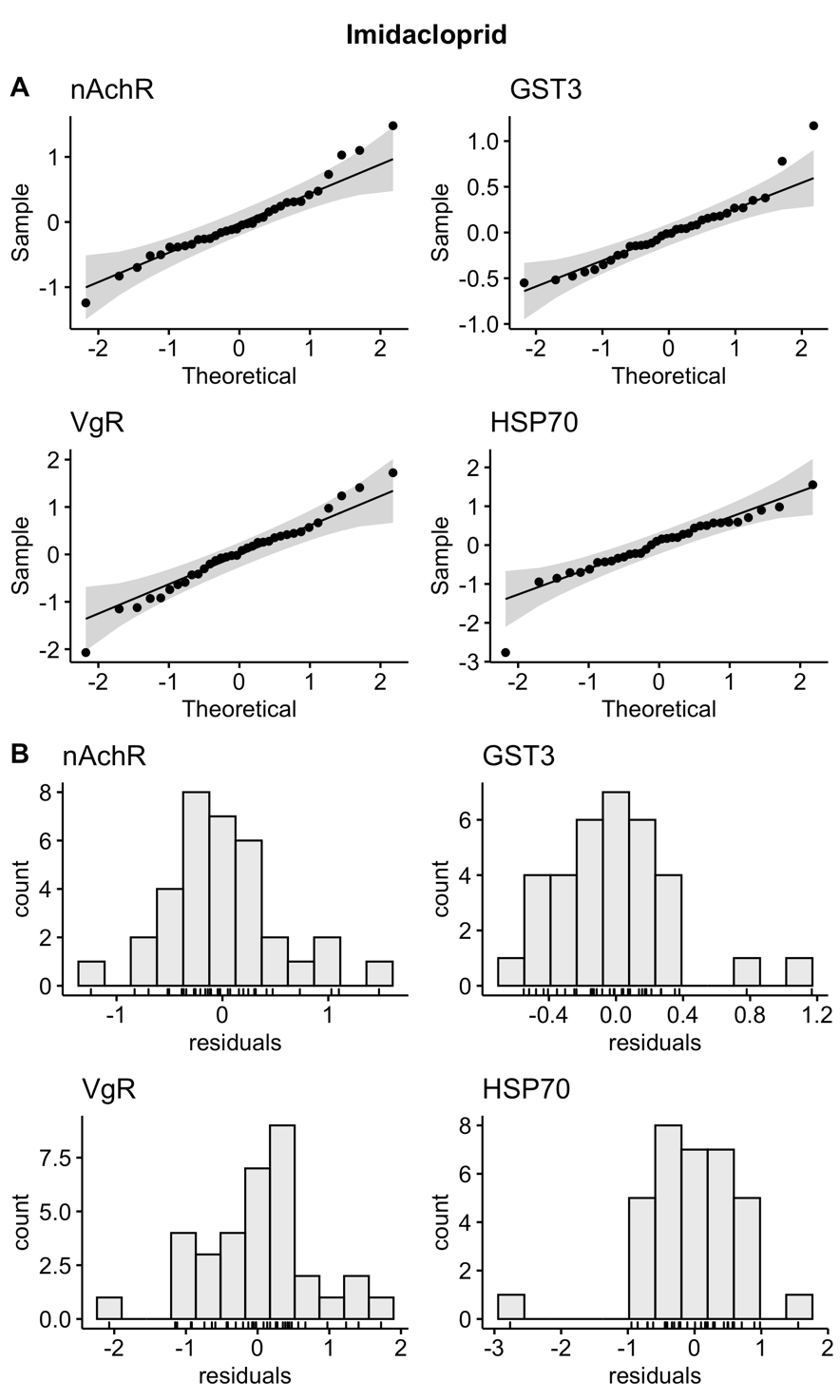


**Figure S-1: Adherence of the residual distribution to homogeneity shown as quantile-quantile plots (A) and histogram frequency plots (B) for all Generalized Additive Models (GAMs) fitted on log2-transformed normalized gene expression measured by qPCR from *Folsomia candida* exposed for 48 hours to imidacloprid and diethyl maleate (DEM) in LUFA 2.2 soil.** Residuals are shown as dots on the quantile-quantile plot panels (A), a solid black line indicates perfect adherence to homogeneity with grey bands indicating 95 % confidence intervals. Residuals are shown as ticks on the x-axis of the histogram frequency plots (B) with their frequency of occurrence indicated by the height of the bars. Each plot shows the result of one primer set, their names are abbreviated above the panels: *nicotinic Acetylcholine Receptor subunit alpha 1* (*nAchR*), *Glutathione-S-Transferase 3* (*GST3*), *Vitellogenin Receptor* (*VgR*) and *Heat Shock Protein 70* (*HSP70*).


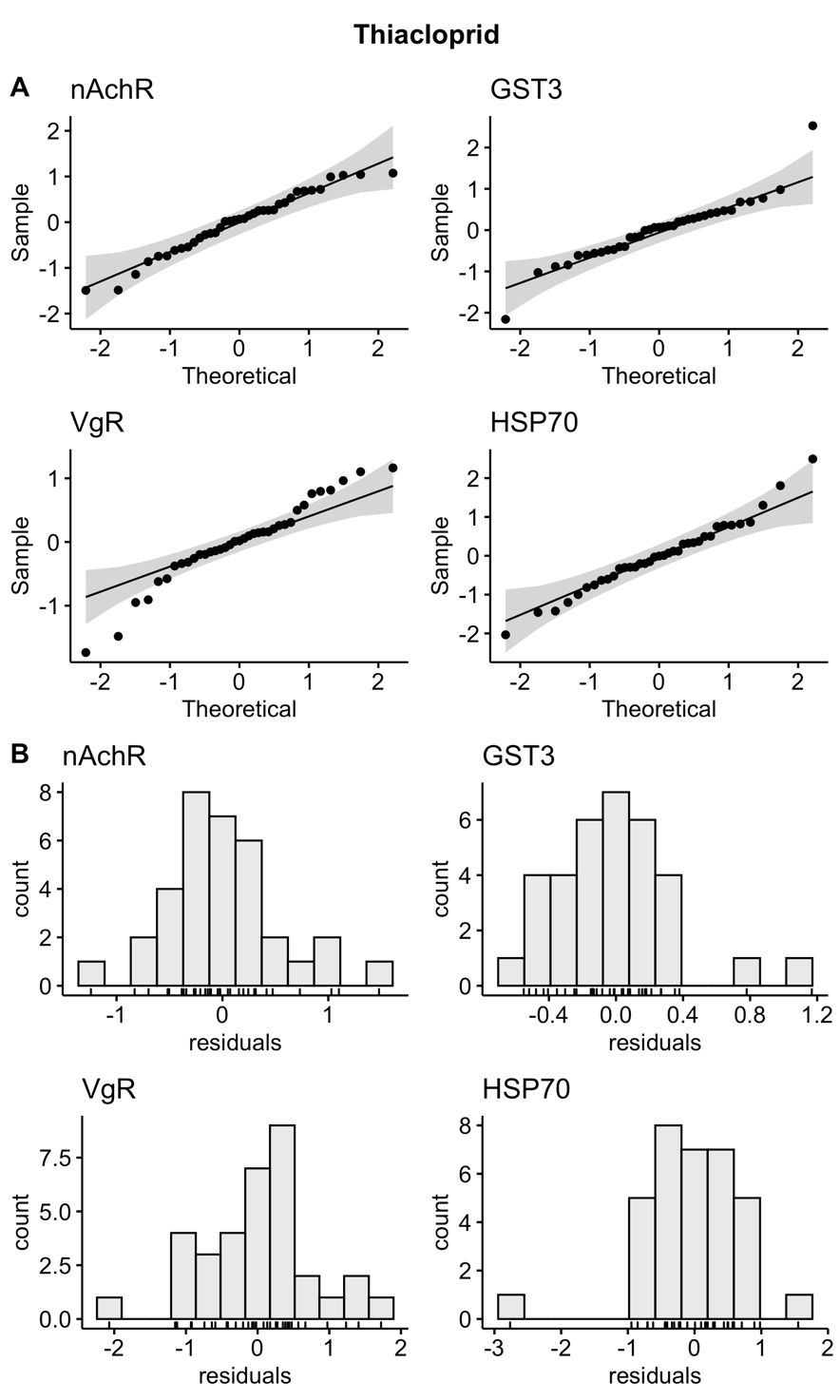


**Figure S-2: Adherence of the residual distribution to homogeneity shown as quantile-quantile plots (A) and histogram frequency plots (B) for all Generalized Additive Models (GAMs) fitted on log2-transformed normalized gene expression measured by qPCR from *Folsomia candida* exposed for 48 hours to thiacloprid and diethyl maleate (DEM) in LUFA 2.2 soil.** Residuals are shown as dots on the quantile-quantile plot panels (A), a solid black line indicates perfect adherence to homogeneity with grey bands indicating 95 % confidence intervals. Residuals are shown as ticks on the x-axis on the histogram frequency plots (B) with their frequency of occurrence indicated by the height of the bars. Each plot shows the result of one primer set, their names are abbreviated above the panels: *nicotinic Acetylcholine Receptor subunit alpha 1* (*nAchR*), *Glutathione-S-Transferase 3* (*GST*3), *Vitellogenin Receptor* (*VgR*) and *Heat Shock Protein 70* (*HSP70*).

# References:

Cunningham, F., Achuthan, P., Akanni, W., Allen, J., Amode, M. R., Armean, I. M., Bennett, R., Bhai, J., Billis, K., Boddu, S., Cummins, C., Davidson, C., Dodiya, K. J., Gall, A., Girón, C. G., Gil, L., Grego, T., Haggerty, L., Haskell, E., … Flicek, P. (2019). Ensembl 2019. Nucleic Acids Research, 47(D1), D745–D751. <https://doi.org/10.1093/nar/gky1113>

OECD (2016). Collembolan reproduction test in soil. Guidelines for Testing Chemicals 232. Organization for Economic Co-operation and Development, Paris.
